# The vital role of constitutive GPCR activity in the mesolimbic dopamine system

**DOI:** 10.1038/tp.2013.130

**Published:** 2014-02-11

**Authors:** F J Meye, G M J Ramakers, R A H Adan

**Affiliations:** 1 Institut du Fer à Moulin, Paris, France; 2 Inserm, UMR-S 839, Paris, France; 3 Université Pierre et Marie Curie, Paris, France; 4 Department of Translational Neuroscience, Brain Center Rudolf Magnus, University Medical Center, Utrecht, The Netherlands

**Keywords:** constitutive activity, dopamine system, GPCR, mood effects, addiction, obesity

## Abstract

The midbrain dopamine system has an important role in processing rewards and the stimuli associated with them, and is implicated in various psychiatric disorders. This system is tightly regulated by various G protein-coupled receptors (GPCRs). It is becoming increasingly clear that these receptors are not only activated by (endogenous) agonists but that they also exhibit agonist-independent intrinsic constitutive activity. In this review we highlight the evidence for the physiological role of such constitutive GPCR activity (in particular for cannabinoid 1, serotonin 2C and mu-opioid receptors) in the ventral tegmental area and in its output regions like the nucleus accumbens. We also address the behavioral relevance of constitutive GPCR signaling and discuss the repercussions of its abolition in dopamine-related psychiatric diseases.

The midbrain dopamine system comprises a neural network critical in processing rewards and their cues.^1^ The importance of its functional integrity is strikingly underlined by some of the serious pathologies associated with its malfunction, such as drug abuse, obesity and depression.^2, 3, 4^ Several G protein-coupled receptors (GPCRs) have a pivotal role in the modulation of the activity of the midbrain dopamine system and its targets. Recent findings show that the *in vivo* activation mechanisms of these receptors go beyond agonist-dependent signaling. Indeed, agonist-independent constitutive GPCR activity (Figure 1) appears to have a vital role in native brain tissue, and interfering with its function can have deleterious effects.^5, 6, 7^ These findings have large repercussions for our understanding of GPCR control of neural networks and likely also for efficacious and safe drug design. The aim of the current review is to describe the role that the constitutive activity of several key GPCRs has in regulating the highly therapeutically relevant midbrain dopamine system.

## GPCR modulation of the mesolimbic dopamine system

**Figure 2:**
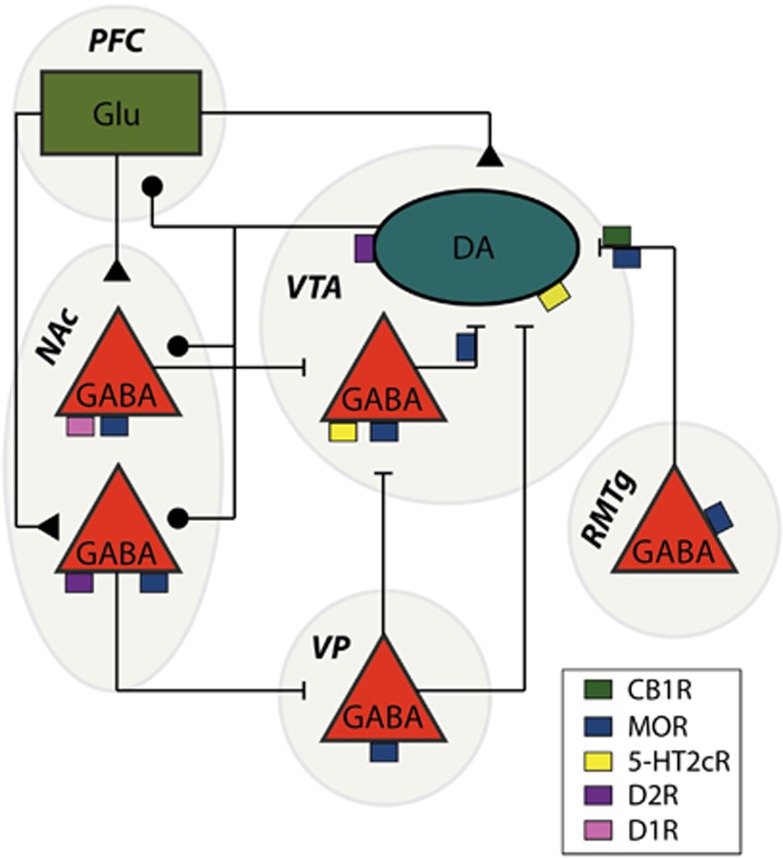
Schematic representation of the main connections of the midbrain dopamine neurons and their control by several key GPCRs. VTA dopamine neurons receive GABAergic inhibition from local GABA neurons, as well as GABA neurons from the rostromedial tegmental nucleus (RMTg). Medium spiny neurons in the NAc receive dopaminergic input from the VTA and project back either monosynaptically (direct pathway) or disynaptically through the ventral pallidum (VP; indirect pathway). The prefrontal cortex (PFC) provides an important glutamatergic (Glu) input to both the medium spiny neurons in the NAc and to neurons in the VTA, while also receiving dopaminergic input itself. Cannabinoid 1 (CB1), dopamine 1 (D1) and 2 (D2), serotonin 2 C (HTR2C) and mu-opioid receptors (MORs) impinge on this network at various levels.

Dopamine signaling patterns are largely dictated by the activity and firing mode of dopamine neurons in the ventral tegmental area (VTA), which project to several structures, including the nucleus accumbens (NAc), ventral pallidum (VP) and the prefrontal cortex (PFC).^1^ Especially the projection to the NAc has an important role in motivated appetitive behavior.^1^ Within the NAc, GABAergic medium spiny projection neurons (MSNs) are divided into those expressing the dopamine 1 receptor (D1R), which directly project back to the VTA (direct pathway), and those (expressing the dopamine 2 receptor (D2R)), which project back disynaptically after first impinging onto the VP.^8^ Excitation of striatal D1R-MSNs is associated with reinforcing behavior, whereas activation of striatal D2R-MSNs has opposite effects.^9^ On top of the important role of these dopamine receptors, the activity of the VTA and its projection targets are modulated by several other GPCRs, some of which have been outlined in Figure 2. In this review we particularly focus on the role of three GPCRs in this neural circuit: the serotonin 2C receptor (HTR2C), the mu-opioid receptor (MOR) and finally the cannabinoid 1 receptor (CB1R). These are the GPCRs for which there is currently compelling evidence that their constitutive signaling contributes to their regulation of the VTA and its projection targets. In the following sections we elaborate on the functional role of the (constitutive) activation of these receptors.

### Constitutively active serotonin 2C receptor: role in dopamine signaling and mood regulation

The G_q_-coupled HTR2C serves an important function in mood regulation. HTR2C activation is anxiogenic (inducing anxiety), whereas interference with HTR2C signaling is anxiolytic (relieving anxiety).^10^ Part of the effects of HTR2C activity on emotional regulation may originate from its role in the mesocorticolimbic networks, which greatly contribute to emotional processing and depression-like behavior.^4,11,12^ Agonist-induced HTR2C activation counteracts dopamine signaling in the NAc.^13^ Instead, blockade of this receptor is associated with reduced effects of cocaine on locomotor activity and reduced motivation for cocaine seeking and drug relapse potential.^14^ Are any of these effects also affected by HTR2C constitutive activity?

In *in vitro* assays HTR2Cs exhibit constitutive activity for two downstream signaling cascades: the G_q_-mediated phospholipase C (PLC) and the (G protein-independent) β-arrestin-dependent ERK1/2 pathways.^15,16^ Rather than this being restricted to such *in vitro* conditions, evidence has emerged that HTR2C constitutive activity regulates dopaminergic signaling in the NAc. Both HTR2C inverse agonists and neutral antagonists increase VTA dopamine neuron firing and dopamine release.^17, 18, 19^ However, the neutral antagonist SB 242084 affects dopamine release to a lesser extent than the inverse agonist SB 206553.^19^ Importantly, the neutral antagonist was able to fully block the inverse agonistic effect of SB 206553 on striatal dopamine release.^19^ This pattern of effects suggests that both endogenous serotonin acting on HTR2Cs, as well as constitutive HTR2C activity, suppress striatal dopamine release.^13,19^

There are indications that alterations in HTR2C constitutive activity are associated with psychiatric diseases like depression, an affliction often linked to a hypofunctional dopamine system.^11,20^ The evidence partly draws on the fact that the HTR2C is a seemingly unique GPCR for which mRNA editing occurs to produce different isoforms. These isoforms exhibit different functional properties, including (but not exclusively) different levels of constitutive activity.^21^ Mice expressing the unedited INI form of HTR2C (with high constitutive activity) exhibit depressive-like behavior in both the forced swim and tail-suspension tests. Contrarily, mice expressing the edited VGV form (with low constitutive activity) show the opposite phenotype in such tasks.^22^ Interestingly, alterations in HTR2C isoforms also occur in brain tissue of patients with depression^23^ and antidepressant treatment alters HTR2C mRNA editing.^24^ Moreover, antidepressants also directly affect HTR2C constitutive activity, with some acting as HTR2C neutral antagonists and others as inverse agonists. The tetracyclic antidepressants mianserin and mirtazapine are HTR2C inverse agonists for both the G_q_-PLC-inositol phosphate and the ERK1/2 pathway. Instead, the selective serotonin reuptake inhibitor (SSRI) fluoxetine and the serotonin antagonist and reuptake inhibitor (SARI) trazodone are neutral antagonists for these HTR2C pathways.^16,25^ Mianserin and mirtazapine lead to large increases in dopamine signaling in the PFC (rather than the NAc), which has been postulated to underlie their antidepressant potential.^26^ Unfortunately, such antidepressant drugs have a broad array of effects on multiple receptors, which makes it difficult to selectively identify the ramifications (beneficial or detrimental) of HTR2C inverse agonism versus neutral antagonism. This clearly calls for studies on the differences between selective HTR2C inverse agonists and neutral antagonists on therapeutically relevant behaviors, which are currently lacking to our knowledge. Such findings do exist for other GPCRs like the mu-opioid (MOR) and cannabinoid 1 receptors (CB1R) however.

### Mu-opioid receptors and addictive behavior

Opioid signaling in the VTA and NAc is triggered by intake of various drugs of abuse.^27, 28, 29^ In the VTA, MOR activation increases firing and burst frequency of dopamine neurons.^30,31^ This effect is mediated by suppression of GABAergic inhibition,^32,33^ but also requires the presence of a glutamatergic tone.^33^ Concomitant with an increase in dopamine levels in the NAc is the direct release of endogenous opioids in this area, which has a critical role in the motivational and hedonic properties of stimuli.^1^

Antagonists for the MOR, such as naltrexone and naloxone, have been propagated to both help treat drug addiction and counteract opiate overdose. Interestingly, these compounds act as inverse agonists at the MOR, when the system has been pre-exposed to opiates.^5^ In such situations these compounds induce strong withdrawal symptoms,^5,6^ likely at least in part by causing hypoactivity of the midbrain dopamine system.^34, 35, 36, 37^ There is now evidence to suggest that the drug withdrawal effects precipitated by MOR inverse agonists like naloxone, are at least partly due to suppression of constitutive MOR activity in the VTA and the (ventral) striatum (see below).

### The relevance of constitutive MOR activity in drug withdrawal symptoms

Evidence for inverse agonistic effects at the MOR in physiological settings is compelling. Inverse MOR agonists reduce constitutive MOR recruitment of G proteins in brain homogenates of mice, but not in MOR knockout mice.^38^ Moreover, constitutively active MORs on GABAergic afferents to VTA dopamine neurons suppress GABAergic transmission in mouse brain slices. The latter was uncovered by the MOR inverse agonist KC-2-009, which itself strongly increased such GABAergic neurotransmission. That effect was not due to interference with endogenous opioid MOR-signaling, as the neutral MOR antagonist CTOP did not affect GABA transmission. CTOP importantly fully blocked the effect of KC-2-009, indicating that the inverse agonistic effect of the latter was MOR-dependent.^32^

Beyond its presumed function in basal regulation regions like the VTA, constitutive MOR activity is modifiable. For instance, it increases after withdrawal from opiate (for example, morphine) treatment. This conclusion is derived from the observation that pretreatment with MOR agonists enhances the effect of MOR inverse agonists, but not of a neutral antagonist, on G protein signaling.^38,39^ Strikingly, pretreatment with morphine can fully reveal inverse agonistic function of certain MOR ligands such as naloxone and naltrexone. This is also true in regions like the midbrain and the striatum. There, morphine pretreatment reveals an inverse agonistic effect of naloxone, but not of neutral antagonist 6β-naltrexol (a naltrexone metabolite), on G protein signaling and adenylyl cyclase activity.^38,40^ Withdrawal from chronic morphine treatment also enhances the effect of MOR inverse agonist KC-2-009, but not neutral antagonist CTOP, on GABAergic inputs to VTA dopamine neurons.^32^

Such findings point towards an adaptive increase in MOR constitutive activity during opiate withdrawal. Potentially this is to partly compensate for the sudden lack of opiate-induced MOR stimulation that the system has grown accustomed to. Interference with constitutive MOR activity, in mesolimbic neural circuitry, may therefore especially have strong effects during states of opiate withdrawal. Indeed, withdrawal symptoms from morphine treatment, such as tremors, jumping and defecation, are precipitated more strongly by MOR inverse agonists than with an equipotent dose of MOR neutral antagonist 6β-naltrexol. This is most likely due to constitutive MOR activity rather than any off-target effect, since 6β-naltrexol attenuated the effects of an MOR inverse agonist on withdrawal symptoms.^40^ Chronic morphine intake, which would enhance constitutive MOR activity, also increases the conditioned place aversion induced by naloxone, but not that induced by MOR neutral antagonists.^6^ In view of these findings in animal models, MOR neutral antagonists may have the potential to provide fewer or less severe withdrawal symptoms than naloxone/naltrexone in humans. Relevant applications in this regard are treatment of opioid overdose and forms of drug addiction. However, it remains to be determined if neutral antagonists are indeed as efficacious as their inverse agonistic counterparts. There is tentative evidence that suggests that neutral antagonists are indeed capable of suppressing drug consumption,^41^ but additional studies are required.

### Cannabinoid 1 receptors: role in food intake, obesity and negative emotions

The endocannabinoid system in the brain facilitates food intake, and its overactivity is implicated in the etiology of obesity.^42,43^ Key neural circuitry underlying these effects includes the hypothalamus and the limbic forebrain, where endocannabinoid levels rise during periods of hunger.^44^ In order to interfere with such endocannabinoid hunger signals, the CB1R inverse agonist rimonabant was marketed to treat obesity in humans. However, it (and similar compounds with it) was ultimately discontinued due to its association with feelings of anxiety and depression in a subgroup of subjects.^45^ A potential site of action for some of the therapeutic, and also the detrimental effects of CB1R inverse agonists, is the mesolimbic circuitry. Activity of dopamine neurons contributes to (food) reward-related behavior,^1,2^ and the hypofunctionality of these neurons has a crucial role in aversive behaviors.^4^

The constellation of effects observed with the CB1R inverse agonist rimonabant could potentially be parsed in effects that are solely due to interference with an endocannabinoid tone, and effects that are due to the additional suppression of constitutive CB1R activity. A prerequisite for this is that the CB1R constitutive activity that is described in heterologous expression systems,^46,47^ also occurs *in vivo* in relevant brain regions. It was recently shown that CB1R constitutive activity occurs in the VTA. In mouse brain slices, the CB1R inverse agonists rimonabant and AM251 increased GABAergic transmission onto VTA dopamine neurons: an effect not observed with two neutral CB1R antagonists O-2050 and NESS0327. In line with the inverse agonistic effect occurring due to suppression of CB1R constitutive activity, pretreatment with NESS0327 fully blocked any subsequent inverse agonistic effect of rimonabant. Similar evidence for CB1R constitutive activity was observed in the basolateral amygdala.^48^ The presence of CB1R constitutive activity in native tissue suggests that drugs like rimonabant can indeed have partially different effects than neutral CB1R antagonists. We now further discuss findings concerning this topic.

### CB1R constitutive activity and effects on hedonic and emotional processing

An important question is whether interference with endocannabinoid signaling at CB1Rs in the brain is sufficient to reduce food intake. If so, then CB1R neutral antagonists and inverse agonists should both be able to exert this therapeutic effect. In animal models, both neutral CB1R antagonists and inverse CB1R agonists lead to comparable reductions in food intake and body weight gain.^48, 49, 50^ Therefore, the suppression of constitutive CB1R activity (on top of the interference with endocannabinoid-CB1R signaling) does not appear to lead to an additional therapeutic effect (although there might be a component for CB1R constitutive activity in the periphery rather than in the brain^51^).

With regard to side effects however, several reports of differences between CB1R inverse agonists and neutral antagonists exist in the domains of malaise, and anxiety- and depression-like behaviors: symptoms that were all reported by a subset of human subjects taking rimonabant.^45^ At higher doses, inverse CB1R agonists can cause a degree of illness, whereas neutral CB1 receptor antagonists appear to lack these side effects.^49,52^ Moreover, CB1R inverse agonists exert CB1R-dependent anxiogenic effects in mice,^53^ which may involve elevations of activity in amygdalar circuitry and hypofunctionality of the dopamine system.^7,48^ Interestingly, the neutral CB1R antagonists AM4113 and NESS0327 do not have these same effects on dopamine neurons and amygdalar circuitry, nor do they appear to raise anxiety.^7,48^ Finally, it is becoming clear that removal of CB1R signaling is associated with depression-like behavior in animal models. CB1R knockout animals, which obviously lack both agonist-dependent and constitutive CB1R signaling, are more susceptible to anhedonic effects of chronic mild stress.^54^ That effect may be particularly mediated by the lack of CB1Rs in dopamine receptive neurons.^55^ Furthermore, there is preclinical evidence that chronic use of the CB1R inverse agonist rimonabant induces a depression-like phenotype on a number of parameters, including more immobility in the forced swim test, reduction in prefrontal serotonin signaling and elevations in cytokine levels.^56^ Other hallmarks of depression are reductions in pleasure (anhedonia) and reductions in motivation and drive (avolition/anergia).^57^ There are indications that CB1R ligands act on animal behaviors relevant to this. CB1R inverse agonists acutely decrease motivation for rewards and (after chronic use) reduce sucrose preference.^48,56,58,59^ It was recently found that the neutral CB1R antagonist NESS0327 does not reduce motivation for sucrose reward and could block such an effect of rimonabant.^48^ Interestingly, other studies showed that neutral CB1R antagonists do reduce operant responding for palatable food on schedules where the required effort to obtain a reward is relatively low.^60,61^ One study also suggested that, as effort costs increase, the potency of neutral antagonists to reduce motivated behavior may decrease.^60^ In an environment where (palatable) food is abundant and easily obtainable at low effort, this may be an appealing pharmacological profile of a drug, as it might reduce low effort (over)consumption, without the risk of general effects on motivated behavior with potential effects on depressive-like behavior.

**Figure 3:**
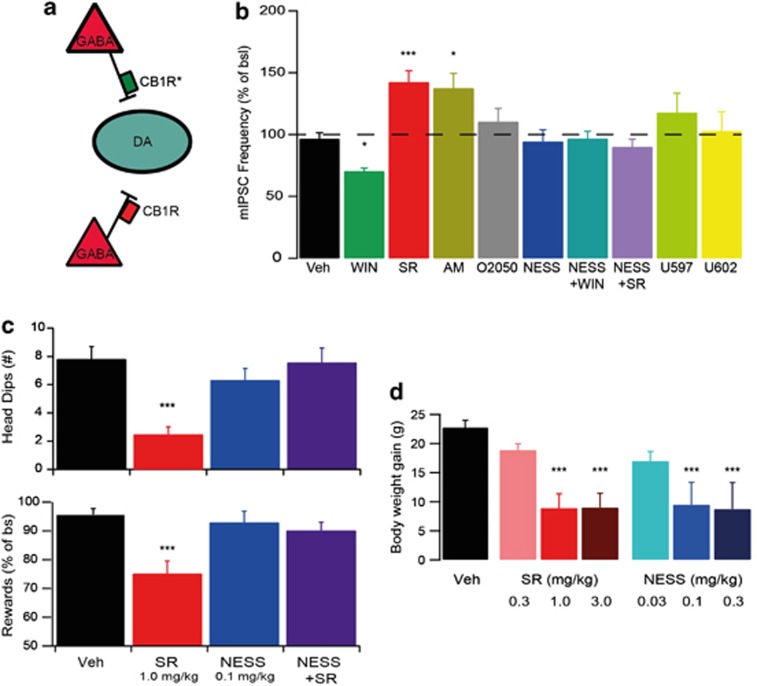
*In vivo* relevance of CB1R constitutive activity. (**a**) Ventral tegmental area dopamine (DA) neurons are regulated by cannabinoid 1 receptor (CB1R)-expressing GABAergic neurons. Some of these CB1Rs (green; CB1R*) are constitutively active, whereas others are not (red, CB1R). (**b**) In mouse brain VTA dopamine neurons, CB1R agonist WIN55,212-2 reduces GABAergic input (miniature inhibitory postsynaptic currents; mIPSCs), whereas the inverse agonists SR141716A (rimonabant) and AM251 increase such GABAergic inhibition. This inverse agonistic effect is presumably by suppressing CB1R constitutive activity, because neutral CB1R antagonists O-2050 and NESS0327 do not affect GABAergic input themselves, whereas the latter does block the effect of both CB1R agonist WIN55,212-2 and SR141716A. Indeed, the effect of the inverse agonists in this slice preparation is not due to the interference with endocannabinoid-CB1R signaling, because indirect agonists URB597 and URB602, which act by preventing the degradation of the endocannabinoids anandamide and 2-Ag respectively, were ineffective in slice preparation. (**c**, top) SR141716A reduces motivation for sucrose reward on a progressive ratio schedule. NESS0327 does not, but does block the effect of SR141716A. (Bottom) Similarly, SR141716A is anxiogenic in the elevated plus maze. NESS0327 is not, although it does block the effect of SR141716A. (**d**) SR141716A and NESS0327 reduce body weight gain to a similar extent. *P<0.05; ***P<0.001.

Overall, these findings suggest that CB1R inverse agonists and neutral antagonists similarly reduce food intake and regulate body weight, while they differ in their propensity to affect anxiety and potentially depression-like behaviors. This preclinical evidence suggests that CB1R neutral antagonists have the potential to be efficacious and safer drugs (for instance for obesity treatment) than the therapeutically discarded CB1R inverse agonists (Figure 3).

## Concluding remarks

It is becoming well established that constitutive activity of GPCRs is not an epiphenomenon of *in vitro* systems but has great physiological relevance. A powerful tool to address such physiological relevance is contrasting the effect of neutral antagonists and inverse agonists to detect qualitative or quantitative differences in responses. If such differential responses are indeed detected, care must be taken to verify that the difference is not simply due to off-target effects of one of the ligands. Helpful control experiments to this end include the attempt to block an inverse agonistic effect with a neutral antagonist, or verifying the absence of an inverse agonistic effect in a system lacking receptor in question. While such approaches are commonly employed in *in vitro* settings, it remains relatively underused in *ex vivo/in vivo* assays, and indeed many outstanding questions about the physiological role of constitutive GPCR activity still need to be addressed (Box 1). The three receptors that were the focus of this review appear to exhibit constitutive activity of GPCRs in regions such as the VTA and NAc. They are likely not the only ones to do so however. There is some level of evidence for constitutive activity of multiple GPCRs that act in mesocorticiolimbic circuitry (Table 1). Sometimes, this is only in the form of *in vitro* assays (for example, the dopamine receptors themselves), but sometimes there are also indications from other tissues or systems that GPCR may exhibit constitutive signaling (for example, the ghrelin receptor) (Table 1).

Current insights hint at the dynamism of constitutive GPCR activity. Instead of providing a fixed amount of background receptor activation, constitutive GPCR activity levels can be tuned by external factors, examples of which include cAMP-mediated MOR phosphorylation^5^ and mRNA editing of HTR2Cs.^21^ Moreover, constitutive (as well as agonist-dependent activation) can be modulated by endogenous inverse agonists, like the hypothalamic agouti-related peptide (AgRP) for the melanocortin 4 receptor,^85^ and the hemoglobin-derived hemopressin that acts as an inverse CB1R agonist in very similar ways as the synthetic inverse agonist rimonabant.^67^ Together these findings suggest that the extent and role of constitutive activity for GPCRs can be subject to modulation and can therefore be tissue-, region- and condition-specific.

The repercussions of the existence of constitutive GPCR activity are also evident on a behavioral level; in particular in relation to therapeutics for psychiatric disorders. If a GPCR population exhibits constitutive activity, it seems a necessary consequence that inverse agonists for such a GPCR will produce stronger (or more) effects than neutral antagonists. The question is whether this will prove to be beneficial (enhanced drug efficacy), detrimental (enhanced risk for side effects) or a combination of both. It is becoming clear that an imbalance in mesocorticolimbic dopamine signaling can have negative consequences, as it is associated with a variety of psychiatric disorders and aversive symptoms.^3,4,11,34,35,37^ Evidently, any pharmacotherapy aimed at remedying a dopamine-related disorder needs to take great care not to ‘overshoot' its rebalancing objective. Preclinical evidence suggests that neutral antagonists for the CB1R and MOR produce fewer side effects than inverse agonists, while it appears that this does not necessarily happen at the cost of reduced efficacy in animal models. It stands to reason that as long as the primary aim of a treatment is to reduce signaling of an endogenous ligand at its GPCR in the midbrain dopamine system, neutral antagonists will be less prone to induce side effects compared to inverse agonists, and may be the primary drug of choice.

## Figures and Tables

**Figure 1:**
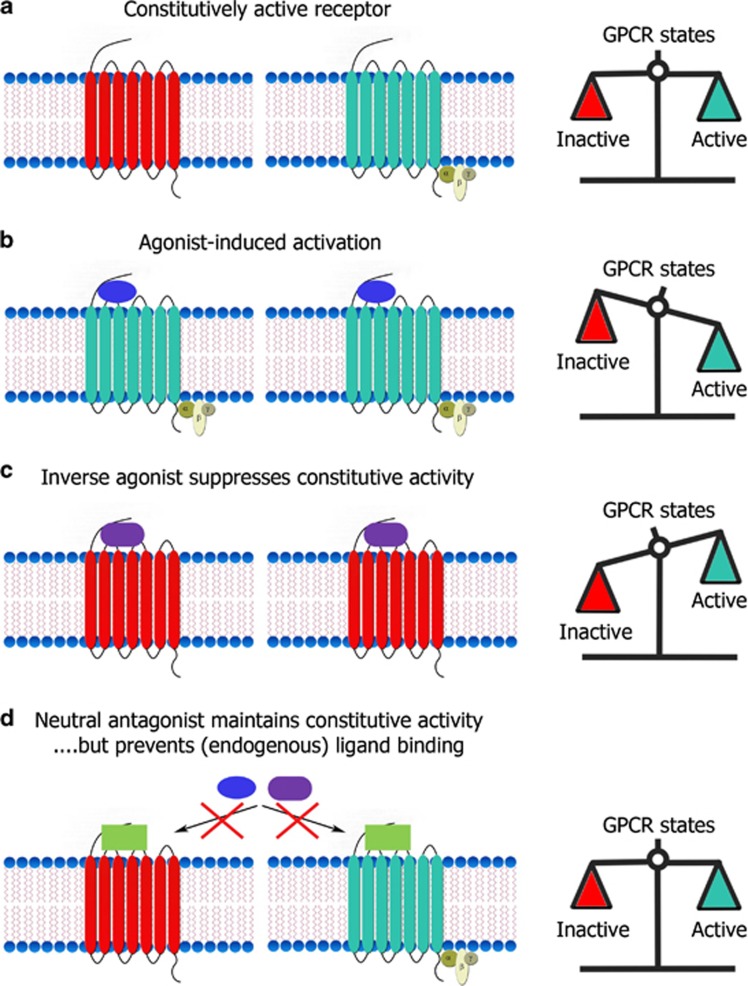
Constitutive GPCR activity and inverse agonism. (**a**) Although G-protein coupled receptors (GPCRs) will typically be in inactive conformations (red) in the absence of an (endogenous) ligand, some spontaneously adopt an active conformation (blue). The extent of this phenomenon makes up the constitutive activity of the receptor population, which is arbitrarily given as 50% (right) in this example purely for illustrative purposes. (**b**) Agonist (blue ellipse) binding to GPCRs shifts the balance toward more active GPCRs, whereas (**c**) an inverse agonists (purple rounded rectangle) shifts the balance towards more inactive receptors. The latter is achieved by a double action: (1) suppression of constitutive GPCR activity and (2) ‘antagonistic' prevention of GPCR activation by (endogenous) agonists. (**d**) In contrast, neutral antagonists (yellow squares) only prevent GPCR activation by (endogenous) agonists, leaving constitutive GPCR activation intact. Notably, neutral antagonists also prevent inverse agonists from suppressing constitutive GPCR activation.

**Table 1:** Constitutive GPCR activity in and beyond the reward system

*Receptor*	*Heterologous systems*	*Relevance in reward system/other systems*
Cannabinoid 1 (CB1R)	↑G_ai/o_ binding^62^ ↑[^35^S]GTPγS^46^ ↓cAMP^49^ ↑MAP kinase activity^63^ ↑GIRK flux^64^ ↓VGCC flux^65^	**Constitutive activity in reward system** ↓GABA on VTA dopamine neurons.^48^ ↑Motivation for rewards.^48^ ↓c-Fos in dorsal and ventral striatum^7^ **Constitutive activity in other systems** ↓Anxiety^7,48^ ↓Glutamate on basolateral amygdala neurons^48^ ↓c-Fos in central amygdala neurons^7^ Potentially ↑GTPγS in several brain structures, although required inverse agonist concentrations were high.^66^ Notably, hemopressin can act as an endogenous inverse agonist for the CB1R.^67^
Mu-opioid (MOR)	↑[^35^S]GTPγS^38,68^ ↓cAMP^68^	**Constitutive activity in reward system** ↓cAMP in striatum^38^ ↓cAMP in midbrain (after morphine treatment)^38^ ↓GABA on VTA dopamine neurons^32^ ↓Morphine withdrawal symptoms^6,68,69^ **Constitutive activity in other systems** ↓cAMP in hippocampus and cortex (after morphine treatment)^38^ ↓Nociception (in β2 arrestin −/− mice)^70^ ↓Nociception after limb injury^71^ ↓Spinal cAMP levels after limb injury^71^ ↓Spinal intracellular Ca^2+^ levels after limb injury^71^
Delta-opioid (DOR)	↑[^35^S]GTPγS^72^ ↑GTPase^73^	**Constitutive activity in reward system** Unknown **Constitutive activity in other systems** Unknown
Serotonin 2a (HTR2A)	↑PLC^74^	**Constitutive activity in reward system** Unknown **Constitutive activity in other systems** ↑Associative learning acquisition^21^
Serotonin 2c (HTR2C)	↑PLC (much more than for HTR2A)^74^ ↑PLA_2_^74^	**Constitutive activity in reward system** ↑Dopamine neuron firing^15^ ↑Striatal dopamine release^15^ **Constitutive activity in other systems** Unknown, although altered RNA-editing of HTR2Cs (which also leads to altered constitutive activity) occurs in the cortex of depressed patients.^23^ Moreover, many antipsychotics also exhibit inverse agonism at this receptor.^74,75^
Dopamine 1/5 (D1R/D5R)	↑cAMP^76,77^ (notably much more for D5R than D1R)	**Constitutive activity in reward system** Unknown **Constitutive activity in other systems** Unknown
Dopamine 2 (D2R)	↑[^35^S]GTPγS^78^ ↓cAMP^78^ ↑GIRK flux^79^	**Constitutive activity in reward system** Unknown **Constitutive activity in other systems** Unknown, although many antipsychotics have inverse agonistic properties at the D2R.^79,80^
Histamine 3 (H3R)	↑[^35^S]GTPγS^81^ ↑Arachidonic acid^81^ ↓cAMP^81^	**Constitutive activity in reward system** Unknown **Constitutive activity in other systems** ↓Histamine from cortical synaptosomes^82^ ↑[^35^S]GTPγS from rat whole brain homogenates^83^ and mouse cortex^82^
Ghrelin receptor (GHSR)	↑IP3^84^ ↑PKC^84^	**Constitutive activity in reward system** Unknown **Constitutive activity in other systems** ↑Bodily growth^84^ ↓Obesity susceptibility^84^ ↑Risk of limbic seizures^84^

Evidence for constitutive activity of several GPCRs that act in the VTA/NAc In this table we have described several key GPCRs that have a role in the regulation of activity in the VTA or NAc. For these receptors we have outlined the *in vitro* evidence for their constitutive activity. Moreover, we have described whether there is evidence for their constitutive signaling within the reward system, or in any other *in vivo* settings. The table is not meant to be exhaustive for all GPCRs that act in the VTA and NAc.

## Box 1. Outstanding questions

• What are the behavioral consequences of suppression of HTR2C constitutive activity versus interference with serotonin signaling by neutral antagonists at this receptor?

• Do other prominent regulatory GPCRs in the mesolimbic dopamine system for which *in vivo* constitutive activity has been described (for example, dopamine, adenosine, ghrelin and melanocortin 4 receptors) exhibit *in vivo* constitutive activity?

• Does variation in *in vivo* constitutive activity of, for instance, CB1Rs and MORs contribute to dopamine-related pathologies?

• Are hemopressin and AgRP actual *in vivo* modulators of CB1R/MC4R constitutive activity beyond being antagonists, and do other such modulators exist for other GPCRs?

• To what extent is constitutive GPCR activity tissue- and/or cell-specific?

• Do constitutively active receptors necessarily engage in canonical signaling *in vivo*, or can they also engage in alternative signaling?

• Is it a general tendency in animal models that neutral antagonists for GPCRs acting in the midbrain dopamine system have fewer unwanted effects, and is this also true in humans?
